# Meeting report for the 1st skin microbiota workshop, boulder, CO October 15-16 2012

**DOI:** 10.1186/1944-3277-9-13

**Published:** 2014-12-08

**Authors:** Jack A Gilbert, Madeleine Ball, Paul Blainey, Martin J Blaser, Brendan JM Bohannan, Ashley Bateman, John Bunge, Maria Gloria Dominguez-Bello, Slava Epstein, Noah Fierer, Dirk Gevers, Tracy Grikscheit, Leila J Hamdan, James Harvey, Curtis Huttenhower, Benjamin Kirkup, Heidi H Kong, Christian Lauber, Katherine P Lemon, Susan V Lynch, Lance Martin, Charlene Mello, Joseph Palma, Roy Parker, Joseph Petrosino, Julia A Segre, Leslie Vosshall, Rui Yi, Rob Knight

**Affiliations:** 1 Argonne National Laboratory, Argonne, IL, and Department of Ecology and Evolution, University of Chicago, Chicago, IL, USA; 2 Department of Genetics, Harvard Medical School, Boston, MA 02115, USA; 3 Broad Institute and MIT Department of Biological Engineering, Cambridge, MA, USA; 4 Department of Medicine, NYU Langone Medical Center, and New York Harbor VA Medical Center, New York, NY, USA; 5 Institute of Ecology and Evolution, University of Oregon, Eugene, OR, USA; 6 Department of Statistical Science, Cornell University, Ithaca, NY, USA; 7 Department of Medicine, New York University, New York, USA; 8 Department of Biology, University of Puerto Rico, Puerto Rico, USA; 9 Department of Biology, Northeastern University, Boston, MA, USA; 10 Department of Ecology and Evolutionary Biology and CIRES, University of Colorado at Boulder, Boulder, CO, USA; 11 The Broad Institute of MIT and Harvard, Cambridge, MA 02142, USA; 12 Division of Pediatric Surgery, Keck School of Medicine, USC, Saban Research Institute, Children’s Hospital Los Angeles, Los Angeles, CA, USA; 13 George Mason University, Prince William Campus, 10900, university Boulevard, MSN 4d4, Manassas, VA 20110, USA; 14 US Army Research Office, Research Triangle Park, NC, USA; 15 Department of Biostatistics, Harvard School of Public Health, Boston, MA, USA; 16 Department of Wound Infections, Walter Reed Army Institute of Research, Silver Spring, MD, USA; 17 Department of Medicine, Uniformed Services University of the Health Sciences, Bethesda, MD, USA; 18 National Cancer Institute, National Institutes of Health, Dermatology Branch, Center for Cancer Research, Bethesda, MD, USA; 19 CIRES, University of Colorado at Boulder, Boulder, CO, USA; 20 Department of Microbiology, The Forsyth Institute, Cambridge, MA, USA; 21 Division of Infectious Diseases, Boston Children’s Hospital, Boston, MA, USA; 22 Department of Medicine, Division of Gastroenterology, University of California San Francisco, 513 Parnassus Ave., S357, San Francisco, CA, USA; 23 Department of Bioengineering, Program in Epithelial Biology, Stanford University School of Medicine and Stanford University, Stanford, CA, USA; 24 US Natick Soldier Center, Natick, USA; 25 Howard Hughes Medical Institute, Boulder, CO, USA; 26 Department of Chemistry & Biochemistry, and BioFrontiers Institute, University of Colorado at Boulder, Boulder, CO, USA; 27 Department of Molecular Virology and Microbiology, Alkek Center for Metagenomics and Microbiome Research, Baylor College of Medicine, Houston, TX, USA; 28 National Human Genome Research Institute, NIH, Bethesda, MD, USA; 29 Howard Hughes Medical Institute, The Rockefeller University, New York, NY, USA; 30 Department of Molecular, Cellular & Developmental Biology, University of Colorado, Boulder, CO, USA

**Keywords:** Skin microbiome, Army, Bioinformatics

## Abstract

This report details the outcome of the 1st Skin Microbiota Workshop, Boulder, CO, held on October 15th-16th 2012. The workshop was arranged to bring Department of Defense personnel together with experts in microbial ecology, human skin physiology and anatomy, and computational techniques for interrogating the microbiome to define research frontiers at the intersection of these important areas. The workshop outlined a series of questions and created several working groups to address those questions, specifically to promote interdisciplinary activity and potential future collaboration. The US Army provided generous grant support and the meeting was organized and hosted by the University of Colorado at Boulder. A primary forward vision of the meeting was the importance of understanding skin microbial communities to improve the health and stealth of US Army warfighters.

## Introduction

The skin is the human body’s largest organ, playing a critical role as the body’s primary barrier against disease and desiccation [1]. The skin is colonized by a wide range of microbes, the roles of which are just beginning to be understood due to advances in DNA sequencing and metagenomics [2]. Rather than being heterogeneous, the skin microbiota varies substantially from body site to body site [3], with “dry”, “sebaceous”, and “moist” sites being a particular driver of the microbiota [4] and a considerable degree of symmetry between the left and right sides of the body [5,6]. The skin microbiota contains substantial microstructure within the layers of the skin, with microbes resident not only on the surface but throughout, particularly in association with pores and glands [2]. Skin microbiota are personal, providing the opportunity to identify the person from which the skin microbial community structure profile originates to a surprising extent, even marking out objects habitually used by a given individual and potentially allowing forensic identification [7]. Strikingly, although the skin microbiota varies more over time than the microbiota in other human body habitats such as the mouth or the gut [5,8], different individuals tend to maintain their separate identities in terms of the skin microbes they carry. Skin microbes are linked to human body odor [9], although the specific pathways involved are only beginning to be understood [10-12] -- in a military context, these odors can give away troop positions, and even affect mosquito attractiveness [13-16].

### Day 1

The first day of the workshop comprised the arrival, primary presentation and discussion forum for the meeting. The meeting was held in the Millennium Harvest House, 1345 Twenty-Eighth St, Boulder, CO 80302, USA, and was attended by 35 people. Each attendee gave a 1-2 minute introduction on who there were, why they were attending, and what they hoped to gain from this meeting. The group comprised many disciplines including biologists, biophysicists, human skin physiology specialists, skin anatomy specialists, medical doctors, US Army and US Navy researchers, microbial ecologists, microbiologists, bioinformaticians, computational biologists, programmers, and mathematicians. This group covered a extremely broad range of expertise, but had a common theme, in that each attendees research has a focus on the interface between microbial consortia and skin physiology.

The meeting started with an introduction and welcome by Rob Knight who organized the meeting and defined the agenda. Wally Buchholz, program manager from the US Army Research Office Life Sciences Division, provided an initial overview of why the Army Research Office was interested in the microbiology of human skin. Wally has subsequently left the office, and Dr. Virginia Pasour has taken over this role. The specific themes put forward in this brief introduction related to the health and stealth of the warfighter in the field. Several potential research targets of interest to the Army were highlighted, including the role of skin microbiota in the attraction of biting insects, the role of microbiota in preventing or causing skin diseases (e.g. fungal infections, spontaneous abscesses, wound infections, etc.), and the role of microbiota in body odor (e.g. do skin microbiota affect how we smell, and can microbiota-produced odors undermine a warfighters capacity to ‘blend in’ to their environment). Then Rob Knight provided a brief but comprehensive assessment of progress and unsolved problems in skin microbial ecology. This talk highlighted the previous work that had been done to characterize the microbial consortia that inhabit skin surfaces, including patterns of spatial and temporal variation and a brief overview of methods for reading out the skin microbiota, and also reiterated specific potential areas of concern such as the role of microbial communities in inhibiting or advancing skin disease, their role in attracting biting insects and their role in human scent. This brief introduction provided an overview for the workshop and promoted the need for interdisciplinary interactions.

Following a brief coffee break, providing an opportunity for interactive discussion, three overview talks provided reviews of skin microbiology from different viewpoints: dermatology, microbial ecology, and molecular microbiology, followed by an overview of the technologies currently used to explore the microbiology of skin. Heidi Kong (National Cancer Institute, USA) began, with an overview talk on the physiology and anatomy of human skin from a dermatological perspective, entitled “More than Skin Deep: An overview of the structure and function of human skin”. This talk highlighted the complexity of the skin. The skin is an essentially multi-laminar organ composed of several cell layers that undergo a continuous process of differentiation producing microscopic and macroscopic structural diversity. The skin provides a range of physiological functions, including a physical permeability barrier, active immunity, protection against disease, wound repair and regeneration, thermoregulation, sensation and appearance. However, the skin can also be subject to a range of dysfunctions, including problems induced by toxins or chemicals, dehydration, skin cancers, autoimmune disorders, viral, bacterial and fungal infections, burns, skin cancer, hyper-/hypothermia, Hansen’s disease (leprosy), vitiligo, photoaging, etc. There followed a detailed overview of the microscopic diversity of skin, which consists of different layers including the stratum corneum, granular layer, spinous layer, and basal layer, cellular components including keratinocytes, Langerhans cells, Merkel cells, melanocytes, resident T cells, and microbes, and appendages including sweat glands, sebaceous glands, hair follicles, and nails [17]. The discussion of skin immunity, and the innate and adaptive immune response in skin, was especially informative. This presentation also described the topographical variability of skin macroscopic structure and morphology across the human body, highlighting specific variability in thickness, and the distribution of hair, eccrine and apocrine sweat glands, and the density of sebaceous glands. All these factors potentially affect the structure and stability of the microbial assemblages that colonize and develop in each region, although the specific influence of each of these factors and the magnitude of the effect remains unknown. Similarly, other host and environmental factors including sexual maturity, aging, genetics, health status, and hygiene, emollients, clothing and climate were also suggested to have a significant impact on microbial assemblage formation, although again research into the relative effects of these factors is largely nascent.

Julie Segre (National Human Genome Research Institute, USA) gave the next overview talk, entitled “Elucidating the diversity of the human microbiome”. This talk described both traditional and new approaches to studying skin microbiology, and how these approaches have changed our perspective of skin microbial ecology in recent years. The medical community has a long history of studying the skin microbiota, and much of this early work was performed in a military context (including Evans et al [18]), Shehadeh and Kligman [19], Leyden et al [20], and US-Army (e.g. during the Vietnam war exploring how a hot and humid climate promotes skin disease and the impact of biting insects on warfighters). Participants found the discussion of the skin microbial biomass fascinating: a swab of the skin can uncover a density of 10,000 microbial cells per square centimeter, but the estimates of density are much higher using a scrape (50,000/cm^2^) or a skin punch (1,000,000/cm^2^). Thus, the vast majority of microbes live beneath the skin surface, although the diversity estimate by the different methods are similar [3]. There followed a discussion of the variability in microbial community composition and assemblage structure between different skin sites, showing oily, dry, and moist crease skin biome classifications to be a primary driver in community structure [4]. The variability among people within a skin site was also shown to be important: this variability can only be fully assessed by performing longitudinal studies of the skin microbial assemblage, especially high resolution temporal studies of the microbiota of the skin of people’s hands [8]. The potential individuality of people’s microbial consortia also suggests a role for forensic classification of personal microbial signatures [7]. The Human Microbiome Project extended our knowledge of the ‘healthy’ skin microbial community demonstrating that major body sites had signature microbial taxa, but with large variation among the 250 individuals surveyed [21]. The important question of where our skin microbiota originates from was also discussed: for example, during birth, vaginal versus cesarean delivery significantly alters the microbial community found in the skin, the oral cavity and the gut of the infant [22], and the establishment of this initial microbiota may influence the succession of the community during the first year of life and beyond. Work on the microbial assemblages associated with atopic dermatitis was also highlighted [23], as were differences in the microbial communities associated with the skin of different global populations [24]. The talk concluded with a discussion of potential future directions for skin microbial research, including the application of shotgun metagenomics to explore the functional dynamics of skin microbial communities (which is currently limited by skin microbial biomass), and the characterization of other microbes other than bacteria, including fungi, viruses, and protists, as well as microfauna such as mites. A long discussion then ensued regarding the technological developments in low-DNA concentration shotgun metagenomic approaches, including Illumina-Nextera sequencing that can make libraries for shotgun approaches with as little as 1-2 nanograms of starting material. It was agreed that these developments are a ‘game-changer’ for the application of functional gene characterization for skin microbial assemblage studies. The use of negative controls was also discussed, especially for skin swabs. This problem can be significant for many studies because microbial DNA can often be amplified from the reagents used to perform DNA extraction and PCR. Therefore, for low biomass samples, we will need to develop appropriate strategies to screen for ‘contaminants’ in sequence data, and/or strategies for removing contaminants from processing reagents.

The final overview talk of the meeting was provided by Joe Petrosino (Baylor College of Medicine) and Curtis Huttenhower (Harvard School of Public Health), entitled “Strategies and technologies for (skin) microbiome studies”. This talk began by discussing the importance of knowing what question is to be addressed, what samples are accessible, what funding level is available, and the need for appropriate benchmarking of new techniques, in order to decide what technology can best be applied in a given project. Similarly, metadata acquisition and appropriate databases are critical for such studies, and for the subsequent re-use of sequence data in later studies and meta-analyses. For human skin, such information as gender, race, ethnicity, age, place of birth, occupation, BMI, vital signs, tobacco use, medical history, medication history, dietary preferences, etc. are all useful parameters to know. The use of the Genomic Standards Consortium’s Minimal Information about Any Sequence (MIxS) standards [25] was highlighted, and the application of the skin-specific environmental packages was discussed. Considerations for sampling and extraction of material were also highlighted, for example the impact of different DNA extraction techniques (e.g. the MoBio PowerSoil DNA extraction kits used in the Human Microbiome Project [21] and Earth Microbiome Project [26] on microbial community profiles was considered an important area for further definition, as well as the practical and financial implications of processing material for thousands to millions of samples. Considerations included ‘do I need to compare my data to other studies?’ , ‘what information can I get from this extracted DNA, e.g. viruses, RNA, or protists?’ , and ‘is my extraction protocol optimized for low biomass?’ Another important consideration is the application of shotgun metagenomics versus amplicon sequencing, and the need to define your question in the selection of these approaches. The talk also highlighted different DNA sequencing technologies (e.g. Illumina, 454 pyrosequencing and Ion Torrent), and discussed the advantages and limitations for each in terms of sequence quality, read length and number of reads generated per run. This section concluded by discussing the potential application and limitations for exploring viral metagenomics and particle profiling in skin samples. Curtis Huttenhower then highlighted some of the computational tools and analytical techniques available for exploring microbial community structure, including QIIME for amplicon sequence data analysis and interpretation, and tools developed by the Huttenhower Lab for performing complementary tasks including tasks related to shotgun metagenomic sequencing (HUMANn for metabolic reconstruction, MetaPhlAn for taxonomic annotation, microPITA and PICRUSt for linking 16S rRNA and metagenomic studies, and LEfSe and MaAsLin for detecting metagenomic biomarkers). Application of these tools will allow substantial new insights into the skin microbiota.

After lunch, we organized into breakout groups to define clusters of topics related to the skin microbiota and opportunities for future research directions, defined by participant interest. Several key clusters of topics emerged.

The first cluster was related to the healthy microbiota: is there a core microbiota within a person’s skin? How do we perform statistical power calculations to determine appropriate cohort sizes for analysis? How many sequences would we need to collect per sample in order to find it? Is a given person’s microbiome unique and/or identifiable over time? What is the relative effect size of specific technical parameters (DNA extraction, sample collection, taxonomy analysis, etc.) vs biological parameters (body site, individual, clinical states such as dermatitis, mosquito attractiveness, etc.?) What is the best way to define what a “healthy” microbial community is, and is this definition general or individual-specific? Are there indicator species that can be used to define health or disease?

The second cluster was related to different host and environmental effects that might affect the microbiota. For example, what are the environmental determinants of the microbiome, including geography and exposures of different kinds, and can we exploit features such as travel that change the microbiome but not the host and/or skin phenotype to understand which components of variation matter and which represent variation that is not biologically significant? How much does the skin microbiota differ in different populations, and how much do conclusions drawn from one population generalize to others? Is there an age at which the microbiome becomes fixed? What controllable host factors, such as, diet or drugs, and uncontrollable host factors, such as host genetics, lead to differences in phenotypes influenced by skin microbiota including odor and mosquito attractiveness? What are the non-pathogenic organisms that interact with the host and the pathobionts doing? How do these non-pathogenic organisms influence succession, e.g. by changing host responses? How does changing the community in one body site (e.g. the gut) trigger changes in the microbiome at another body site (e.g. the skin) through systemic factors, treatment, immune responses, or other factors? What is the effect of different antimicrobial technologies (e.g. the broad-spectrum antimicrobials used in uniforms) – or, more generally, extrinsic events that do change the microbiota and could perhaps be used to drive a microbiota in a specific direction? Does antimicrobial use increase microbial migration rates? What influence do topical treatments (e.g. antibiotic creams) have?

The third cluster was related to technical challenges. These included questions about how can we better measure microbial biomass? Are there other key technical limitations that, if overcome, would increase our ability to answer specific questions? What is the replication rate of skin microbes, and where on the body and within the skin is most of this replication occurring, e.g. are there source/sink dynamics? Which questions should be answered in animal models, tissue models, or other culture models, and which animal models are most appropriate in order to generalize the results to humans? Is skin unique relative to other body habitats because most of the dominant bacteria are culturable?

Finally, we agreed that several additional questions were interesting, but there was less support from the group for discussing those questions during the workshop. These questions included the question of how we can get better annotations, especially functional annotation of genes given that 2/3 of genes in a typical bacterium are uncharacterized at present, and better reference phylogenies for identifying marker genes at the species level? What is the prevalence of CRISPRs, how common are CRISPRs across the skin within and between subjects and sites, and what does the mobile gene content (including plasmids and viruses) look like? Does horizontal gene transfer occur on the skin, and if so how do rates compare to rates at other body sites? Should studies try to cover the whole skin, or focus on specific body sites (and, if the latter, which body sites?)

### Day 2

The second day consisted of reports from the working groups, and further discussion around more focused topics aimed at delineating possible future collaborations and research programs.

There was an extensive discussion about biomass measurement, and other technological developments that could advance the field. The participants agreed that although improved measurement of biomass would be useful (especially in terms of converting information about relative abundance of organisms to absolute abundance, which would for example allow us to distinguish between a bloom in one organism versus a decrease in other organisms), in general, the barriers to advancing the field stem not from technical limitations but rather from lack of access to samples or subjects. Because many of the techniques are new, a large number of obvious questions have not been addressed and there is substantial opportunity for amassing new knowledge rapidly.

In the first round of working group reports, there was substantial agreement from those reporting (Curtis Huttenhower, Katherine Lemon, Charlene Mello, Jack Gilbert, and Benjamin Kirkup) on the major issues. Soldiers are especially good potential participants in microbiome studies due to their ability to control many factors not controllable in the population in general, including housing, diet, drugs, antimicrobials, and many lifestyle factors.

In addressing environmental determinants of the microbiome, Army personnel provide several unique opportunities. These include training cohorts who are brought together from diverse regions into a single area, individuals pre- and post-deployment, and, potentially, monozygotic and dizygotic twins (although twins may not be specifically tracked by current Army information systems). Siblings would also be useful if twins are not available. Longitudinal studies could also determine whether any homogenization of the skin microbiota occurs primarily through transfer of microbes or from equilibration of groups that are present beforehand but at different abundance. Another interesting possibility is comparing skin in situations where modern HVAC systems are employed to more primitive deployment sites. Some key questions include whether there are specific taxa that act in an equilibrium vs non-equilibrium manner, and whether it is possible to discriminate organisms that live on the skin specifically because it is a favorable environment for them versus those that can survive there despite the harsh conditions.

In addressing controllable and uncontrollable host factor effects, one intriguing possibility, though perhaps requiring additional technology development, is skin immunotyping. Surveys of skin microbiota during immunosuppression, either systemic or local (e.g. with topical corticosteroids) could be interesting in this respect. Studies could also be conducted examining change in the microbiota with bathing and other hygiene practices, which could also inform us about the resilience and dynamics of the skin microbiota to different kinds of perturbations. The role of diet in affecting the skin microbiota has been understudied, and the combination of food frequency questionnaires and/or controlled feeding over different periods, combined with sampling both of the skin and the gut, could be informative in this respect. Especially with intravenous feeding, skin samples could be correlated with an exact vitamin and nutritional profile. For subjects undergoing surgery, especially for appendectomies and hernias, which are frequent, or during wound healing, it would be interesting to test whether specific microbes correlate with different responses (although some of these questions might be better answered in animal models). Cross-population components could be integrated into many of these studies to test whether subject stratification is necessary.

One especially interesting possibility would be the creation of an “Army Microbiome Project” -- the initial entry training for combat troops is 13 weeks, so recruiting four classes annually for two years would provide substantial replication. Similar types of studies over longer periods, e.g. the 4-year periods of the Naval Academy or allowed for graduate work, could also be interesting. These studies could cross multiple body sites, including but not necessarily limited to skin and gut, and could track location, housing parameters, diet, medication, physical activity, biometrics, etc. The most efficient use of samples would be in a stratified design in which a subset of subjects is sampled very frequently (e.g. daily), and a subset at longer intervals; similarly, a small number of subjects could be explored in depth, and a larger number at just a few habitats. This would provide substantial cost-benefit in basic science from controlled longitudinal observation, and could be complemented with more translational work on controlled wound healing in hospital and/or animal settings. Correlating blood or urine metabolism to the microbiome, and understanding how to perform predictive modeling of the skin microbiome (or of synthetic communities), could also be especially useful for determining mechanisms and for understanding how changes in odor could relate from changes in metabolites. Single-cell approaches could also be useful, especially in conjunction with strain collections.

After the initial reports, more specific working groups were then formed to explore more detailed topics. The reports from these working groups highlighted several additional issues.

Maria Gloria Dominguez-Bello from the University of Puerto Rico and the New York University Medical Center reported for her group that the army provides access to an ethnically diverse cohort of healthy, young subjects, providing clear opportunities to track whether these groups converge. One interesting question is the right phylogenetic level to look at in order to test convergence (which could include whole-genome studies from single cells or cultures). Testing whether invasion during co-housing is higher in people who have been on antibiotics, which might destabilize the skin community, could also be interesting, as could testing whether changes in the skin microbiota during deployment could pinpoint an environmental cause for some of the differences in the microbiota seen in cross-sectional studies without migration.

Leslie Vosshall from Rockefeller University noted the clear links that have been demonstrated among mosquito biology, volatile organic compounds, and the skin microbiota. However, the microbiota inhabiting the mosquitoes themselves, and possible effects on insect behavior (as has been demonstrated in Drosophila), are largely unknown. Human attractiveness to mosquitoes is stable over extended periods (years), suggesting possibilities for exploring which aspects of the skin microbiota are similarly stable and could explain these differences. Effects of ethanol wipes and of soap, and of dietary factors that are widely believed by the public to reduce mosquito attractiveness including garlic, vitamin B12, and bananas, have largely not been studied with adequate controls and sample sizes.

Madeline Ball from the Personal Genome Project at Harvard discussed the topic of personal identifiability: what level of analysis is required in order to find unique and personally or regionally identifying signatures? For example, could a handshake transfer enough microbes to confer a unique signature? How do we characterize the stable and the malleable components of the microbiota? Similarly, do either the stable or the malleable components allow specific predictions to be made about invasion of a given person’s microbiota by new microbes?

Martin Blaser from New York University discussed the profound alterations that humanity has wrought on the microbiome with antibiotics, antiseptics, and antimicrobials. There are several important opportunities for research in this area, including examining long-term antibiotic users as “natural experiments” on the effects of antibiotics on the microbiome, perturbation experiments that can be performed in mice and in other animal models, the role of horizontal gene transfer in the skin microbiota and whether antibiotics promote transfer by mechanisms including activating the SOS response, the use of topical treatments as defined perturbations for assessing the magnitude and dynamics of microbial responses over time, the importance of considering the non-bacterial components of the skin microbiota, testing of the effects of the silver nanoparticle socks being widely deployed by the Army on the skin microbiota, and the ability of antibiotics to affect phenotypes such as odors, colonization by harmful microbes, and perhaps even mosquito attractiveness.

### Wrap-up

The participants largely agreed on the following points: many of the technologies for understanding the skin microbiota are now sufficiently mature that substantial progress can be made with existing technologies rather than waiting for additional technological developments (one exception being improved methods for quantifying microbial biomass). Contamination with human DNA is challenging for shotgun metagenomic studies (as opposed to 16S rRNA-based studies, which are largely unaffected), although it may be more effective to screen sequences computationally as sequencing continues to decrease in price rather than to develop new protocols, as sequencing is not the bottleneck. The Army provides unique advantages in terms of providing access to well-controlled, ethnically diverse, and potentially enthusiastic volunteers with outstanding follow-up, and a number of exciting longitudinal studies of the effects of co-housing, diet, and antimicrobials on the microbiome. The effects of the microbiome on topics ranging from mosquito attractiveness to wound healing could be explored fruitfully in this context.

## Competing interest

The authors declare that they have no competing interests.

## Authors’ contributions

JAG, MB, PB, MJB, BJB, AB, JB, MGD-B, SE, NF, DG, TG, LJH, JH, CH, BK, HHK, CL, KPL, SVL, LM, CM, JP, RP, JP, JAS, LV, RY, and RK all contributed to the writing and editing of the manuscript. All authors read and approved the final manuscript.

## References

[B1] Segre JA Epidermal barrier formation and recovery in skin disorders J Clin Invest 2006 116 1150 1158 10.1172/JCI28521 16670755 PMC1451215

[B2] Grice EA Segre JA The skin microbiome Nat Rev Microbiol 2011 9 244 253 10.1038/nrmicro2537 21407241 PMC3535073

[B3] Grice EA Kong HH Renaud G Young AC Bouffard GG Blakesley RW Wolfsberg TG Turner ML Segre JA NISC comparative sequencing program A diversity profile of the human skin microbiota Genome Res 2008 18 1043 1050 10.1101/gr.075549.107 18502944 PMC2493393

[B4] Grice EA Kong HH Conlan S Deming CB Davis J Young AC Bouffard GG Blakesley RW Murray PR Green ED Turner ML Segre JA NISC Comparative Sequencing Program Topographical and temporal diversity of the human skin microbiome Science 2009 324 1190 1192 10.1126/science.1171700 19478181 PMC2805064

[B5] Costello EK Lauber CL Hamady M Fierer N Gordon JI Knight R Bacterial community variation in human body habitats across space and time Science 2009 326 1694 1697 10.1126/science.1177486 19892944 PMC3602444

[B6] Egert M Schmidt I Höhne H-M Lachnit T Schmitz RA Breves R rRNA-based profiling of bacteria in the axilla of healthy males suggests right-left asymmetry in bacterial activity FEMS Microbiol Ecol 2011 77 146 153 10.1111/j.1574-6941.2011.01097.x 21428987

[B7] Fierer N Lauber CL Zhou N McDonald D Costello EK Knight R Forensic identification using skin bacterial communities Proc Natl Acad Sci U S A 2010 107 6477 6481 10.1073/pnas.1000162107 20231444 PMC2852011

[B8] Caporaso JG Lauber CL Costello EK Berg-Lyons D Gonzalez A Stombaugh J Knights D Gajer P Ravel J Fierer N Gordon JI Knight R Moving pictures of the human microbiome Genome Biol 2011 12 R50 10.1186/gb-2011-12-5-r50 21624126 PMC3271711

[B9] Leyden JJ McGinley KJ Hölzle E Labows JN Kligman AM The microbiology of the human axilla and its relationship to axillary odor J Invest Dermatol 1981 77 413 416 10.1111/1523-1747.ep12494624 7288207

[B10] Natsch A Derrer S Flachsmann F Schmid J A broad diversity of volatile carboxylic acids, released by a bacterial aminoacylase from axilla secretions, as candidate molecules for the determination of human-body odor type Chem Biodivers 2006 3 1 20 10.1002/cbdv.200690015 17193210

[B11] Troccaz M Borchard G Vuilleumier C Raviot-Derrien S Niclass Y Beccucci S Starkenmann C Gender-specific differences between the concentrations of nonvolatile (R)/(S)-3-methyl-3-sulfanylhexan-1-Ol and (R)/(S)-3-hydroxy-3-methyl-hexanoic acid odor precursors in axillary secretions Chem Senses 2009 34 203 210 19147808 10.1093/chemse/bjn076

[B12] Barzantny H Schröder J Strotmeier J Fredrich E Brune I Tauch A The transcriptional regulatory network of Corynebacterium jeikeium K411 and its interaction with metabolic routes contributing to human body odor formation J Biotechnol 2012 159 235 248 10.1016/j.jbiotec.2012.01.021 22342369

[B13] Verhulst NO Beijleveld H Knols BG Takken W Schraa G Bouwmeester HJ Smallegange RC Cultured skin microbiota attracts malaria mosquitoes Malar J 2009 8 302 10.1186/1475-2875-8-302 20017925 PMC2804688

[B14] Verhulst NO Takken W Dicke M Schraa G Smallegange RC Chemical ecology of interactions between human skin microbiota and mosquitoes FEMS Microbiol Ecol 2010 74 1 9 10.1111/j.1574-6941.2010.00908.x 20840217

[B15] Verhulst NO Andriessen R Groenhagen U Bukovinszkiné Kiss G Schulz S Takken W van Loon JJA Schraa G Smallegange RC Differential attraction of malaria mosquitoes to volatile blends produced by human skin bacteria PloS One 2010 5 e15829 10.1371/journal.pone.0015829 21209854 PMC3012726

[B16] Verhulst NO Qiu YT Beijleveld H Maliepaard C Knights D Schulz S Berg-Lyons D Lauber CL Verduijn W Haasnoot GW Mumm R Bouwmeester HJ Claas FHJ Dicke M van Loon JJA Takken W Knight R Smallegange RC Composition of human skin microbiota affects attractiveness to malaria mosquitoes PloS One 2011 6 e28991 10.1371/journal.pone.0028991 22216154 PMC3247224

[B17] Kong HH Skin microbiome: genomics-based insights into the diversity and role of skin microbes Trends Mol Med 2011 17 320 328 10.1016/j.molmed.2011.01.013 21376666 PMC3115422

[B18] Evans CA Smith WM Johnston EA Giblett ER Bacterial flora of the normal human skin J Invest Dermatol 1950 15 305 324 10.1038/jid.1950.45 14779047

[B19] Shehadeh NH Kligman AM The effect of topical antibacterial agents on the bacterial flora of the axilla J Invest Dermatol 1963 40 61 71 13977054 10.1038/jid.1963.10

[B20] Leyden JJ McGinley KJ Mills OH Kligman AM Age-related changes in the resident bacterial flora of the human face J Invest Dermatol 1975 65 379 381 10.1111/1523-1747.ep12607630 1176788

[B21] Huttenhower C Gevers D Knight R Abubucker S Badger JH Chinwalla AT Creasy HH Earl AM FitzGerald MG Fulton RS Giglio MG Hallsworth-Pepin K Lobos EA Madupu R Magrini V Martin JC Mitreva M Muzny DM Sodergren EJ Versalovic J Wollam AM Worley KC Wortman JR Young SK Zeng Q Aagaard KM Abolude OO Allen-Vercoe E Alm EJ Alvarado L Structure, function and diversity of the healthy human microbiome Nature 2012 486 207 214 10.1038/nature11234 22699609 PMC3564958

[B22] Dominguez-Bello MG Costello EK Contreras M Magris M Hidalgo G Fierer N Knight R Delivery mode shapes the acquisition and structure of the initial microbiota across multiple body habitats in newborns Proc Natl Acad Sci U S A 2010 107 11971 11975 10.1073/pnas.1002601107 20566857 PMC2900693

[B23] Oh J Conlan S Polley EC Segre JA Kong HH Shifts in human skin and nares microbiota of healthy children and adults Genome Med 2012 4 77 10.1186/gm378 23050952 PMC3580446

[B24] Blaser MJ Dominguez-Bello MG Contreras M Magris M Hidalgo G Estrada I Gao Z Clemente JC Costello EK Knight R Distinct cutaneous bacterial assemblages in a sampling of South American Amerindians and US residents ISME J 2013 7 85 95 10.1038/ismej.2012.81 22895161 PMC3526177

[B25] Yilmaz P Kottmann R Field D Knight R Cole JR Amaral-Zettler L Gilbert JA Karsch-Mizrachi I Johnston A Cochrane G Vaughan R Hunter C Park J Morrison N Rocca-Serra P Sterk P Arumugam M Bailey M Baumgartner L Birren BW Blaser MJ Bonazzi V Booth T Bork P Bushman FD Buttigieg PL Chain PSG Charlson E Costello EK Huot-Creasy H Minimum information about a marker gene sequence (MIMARKS) and minimum information about any (x) sequence (MIxS) specifications Nat Biotechnol 2011 29 415 420 10.1038/nbt.1823 21552244 PMC3367316

[B26] Gilbert JA Meyer F Jansson J Gordon J Pace N Tiedje J Ley R Fierer N Field D Kyrpides N Glöckner F-O Klenk H-P Wommack KE Glass E Docherty K Gallery R Stevens R Knight R The earth microbiome project: meeting report of the “1 EMP meeting on sample selection and acquisition” at Argonne National Laboratory October 6 2010 Stand Genomic Sci 2010 3 249 253 10.4056/aigs.1443528 21304728 PMC3035312

